# Influence of Levan on the Thermally Induced Gel Formation of β-Lactoglobulin

**DOI:** 10.3390/gels8040228

**Published:** 2022-04-07

**Authors:** Christoph S. Hundschell, Juliane Brühan, Theresa Anzmann, Reinhard Kohlus, Anja M. Wagemans

**Affiliations:** 1 Department of Food Colloids, Institute of Food Technology and Food Chemistry, Technical University Berlin, Straße des 17. Juni 135, 10623 Berlin, Germany; juliane.bruehan@gmx.de; 2 Department of Process Engineering and Food Powders, University of Hohenheim, Garbenstraße 25, 70599 Stuttgart, Germany; theresa.anzmann@uni-hohenheim.de (T.A.); r.kohlus@uni-hohenheim.de (R.K.)

**Keywords:** levan, β-lactoglobulin, gel, phase separation, rheology

## Abstract

In this study, the influence of levan on the phase behavior and the thermally induced gelation of the mixed β-lactoglobulin—levan gels as a function of polymer content, molecular weight and ionic strength was characterized. For this purpose, rheology was used to study the mechanical properties of the gels and the water binding of the network structure was investigated by time domain nuclear magnetic resonance. Phase behavior and network type were analyzed by optical observation and electron microscopy. Levan enhanced the aggregation and gel formation of β-lg due to segregative forces between the polymer species. Segregation was caused by the excluded volume effect and was more pronounced at lower ionic strength, higher levan contents and higher levan molecular weights. The presence of levan increased the water binding of the gel networks. However, this effect decreased with increasing levan content. At high ionic strength and high levan content, phase separated gels were formed. While segregative forces enhanced network formation, and therefore, increased the gel strength of mixed gels at low ionic strength, levan had also antagonistic effects on the network formation at high ionic strength and high polymer contents.

## 1. Introduction

To predict the structure and functionality of foods as complex multiphase systems, the understanding of the molecular interactions between the two main types of food biopolymers—polysaccharides and proteins—is of central importance, and therefore, part of ongoing research [1,2,3,4,5]. In systems consisting of two different polymer species, co-solubility, complexation, or thermodynamic incompatibility may occur, depending on the molecular interactions [6]. Co-solubility of two polymers is rare since most polymers interact attractively or repulsively [7]. Attractive interactions cause the formation of soluble complexes or complex coacervation. Segregative phase separation occurs in thermodynamic incompatible systems when the phase separation threshold is exceeded [8]. This type of phase behavior occurs when both polymers interact repulsive or both polymers differ in their affinity for the solvent [6].

In polymer mixtures in which one or both polymers can form a gel, the formation of different networks is possible. An interpenetration network is rarely formed. Here, both polymers must be able to form a gel with the resulting networks of both polymers being independent of each other [9]. If dissimilar polymers bind to each other due to associative interactions, coupled networks are formed [10]. In coupled networks, the intermolecular adhesion zones between both kinds of polymers enable the gel formation for polymers that individually would not form a gel [11]. The most common network types are phase-separated networks, which are formed between dissimilar polymer species that repel each other [10]. In addition, phase-separated networks are formed when the critical gelation concentration is greater than the phase-separation threshold, and therefore, phase separation occurs before or during gelation [12]. Swollen networks are gels in which only one polymer is involved in the formation of the network, while the other polymer is dissolved and/or homogeneously dispersed over the network [13]. This type of gel can be formed in co-soluble systems or thermodynamic incompatible systems below the phase separation threshold. The excluded volume effect, which leads to a local increase in concentration, can enhance the gelation of the network-forming component in this kind of gel. This effect arises from a reduction of the excess entropy of mixing since different polymer species do not have access to the volume occupied by the other species [10].

A well-known and established model protein is β-lactoglobulin (β-lg). It is a globular protein and the main protein component of whey. It has a molecular weight of 18.4 kDa and a hydrodynamic radius of about 2 nm [14,15]. The ability of whey proteins to form a gel is exploited in many food products. The gelation of β-lg can be induced by heating, cold setting, or high pressure [16,17,18]. Heat-induced gelation of β-lg occurs in two steps. Firstly, heating causes denaturation and consequently refolding of the protein. Secondly, aggregation of the proteins enables network formation [15]. The structure of the formed network depends largely on the electrostatic interactions between the protein molecules, and therefore, on the pH value and ionic strength [19]. Far off the isoelectric point and at low ionic strength, when electrostatic repulsion dominates, fibrillar gels are formed. Fibrillar gels have a transparent appearance and the diameter of the gel strands is in the nm range [20]. At a high ionic strength or near the isoelectric point, opaque particle gels with gel pores in the µm range are formed [21]. The presence of polysaccharides in β-lg gels can modify the resulting network. The addition of polysaccharides can antagonistically or synergistically modify the structure and properties of β-lg gels depending on the chemical structure of the polysaccharide, the mixing ratio, and the gelation mechanism [9,22,23].

Levan is a β-2,6 linked fructan that is produced by many food-grade starter cultures [24,25,26,27,28]. It occurs naturally in fermented foods, such as sourdough bread or kefir [29,30]. It has prebiotic properties; it is a source of dietary fiber and it can improve the structural properties of foods [31,32,33,34,35,36]. Its macromolecular and rheological properties are strongly dependent on the molecular weight [37]. In an aqueous solution, low molecular weight levan has a random coil structure, low viscosity, and Newton-like flow behavior. High molecular weight levan has a compact spherical structure. It causes high viscosity and shear thinning behavior, both being more pronounced with increasing molecular weight. From a content of 5 wt%, the elastic properties of high molecular weight levan predominate the viscous properties and the dispersion shows a gel-like behavior [1,38,39]. In a previous study, we reported repulsive pair interactions due to the excluded volume effect in dilute mixtures of native β-lg and levan [1]. These interactions seem to be more pronounced at a higher molecular weight of levan, while the ionic strength has only a minor influence. In contrast to the pair interactions, the predicted phase behavior was mainly affected by electrostatic interactions, and therefore, by ionic strength. At low ionic strength, the electrostatic repulsion counteracts the accumulation of the β-lg in one phase, and therefore, phase separation is less likely. The opposite happens at high ionic strength, where attractive interactions between the protein molecules seem to facilitate phase separation.

Due to its unique physicochemical properties, levan is a promising polysaccharide to enhance the nutritional and techno-functional quality of food systems. However, for its targeted use in food systems, interactions with other biopolymers, and particularly with proteins, must be established. For this purpose, we investigated the influence of levan on the phase behavior and the heat induced gelation of the model protein β-lg. Two different ionic strengths were chosen to obtain either a fibrillar or particle β-lg gel. To ensure gel formation—even at a low ionic strength—protein contents of 10 wt% were chosen. In addition, the content and molecular weight of levan were varied as it directly affects the phase behavior and network formation of the mixed gels. Based on the interactions in dilute systems, we hypothesize the following:

- Heat-induced aggregation of β-lg enhances the segregative forces between β-lg and levan, due to the excluded volume effect.
- A higher levan content and molecular weight promotes phase separation between β-lg and levan, and therefore, the formation of phase-separated networks.
- A higher ionic strength promotes phase separation since the reduced electrostatic repulsion between the β-lg molecules facilitates the accumulation of the protein.

## 2. Results and Discussion

### 2.1. Heat Induced Gelation

**Figure 1:**
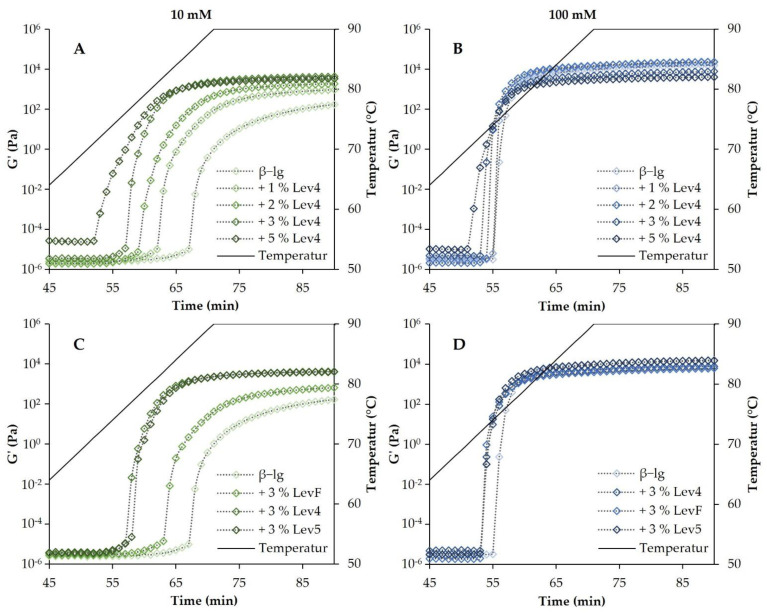
Development of the storage modulus in the temperature sweeps of gels containing different contents of Lev4 (panels (**A**,**B**)), different molecular weights (panels (**C**,**D**)) at 10 mM NaCl (**left** panel) and 100 mM NaCl (**right** panel). The temperature ramp ranged from 20 °C to 90 °C. Shown is the range from 45 °C to 90 °C and the holding phase at 90 °C, where gelation takes place.

**Figure 2:**
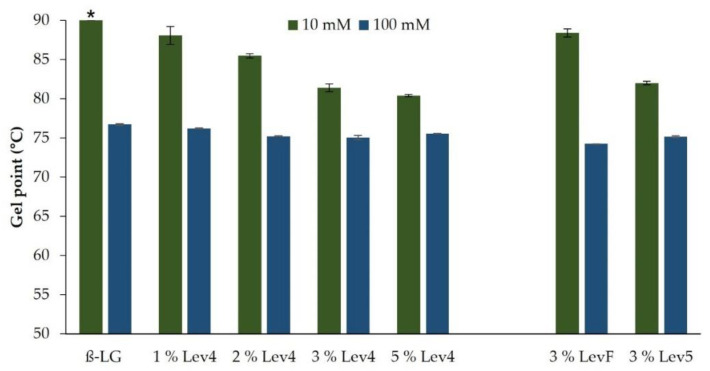
Gel point temperature of samples containing different Lev4 contents (**left** side) and different molecular weights of levan (**right** side). * sample gelled after a holding time of 2.9 ± 0.9 min at 90 °C.

The temperature sweeps of β-lg gels and mixed gels with varying contents of the high molecular weight levan Lev4 (2.0 × 10^8^ Da) and salt concentration are shown in Figure 1. The gelation of β-lg varied considerably with the salt concentration. For the 10 mM β-lg gels, the onset of gelation (initial increase of the storage modulus G′) occurred later and more time was needed to reach a constant value of G′ compared to the 100 mM β-lg gels. For the 100 mM, β-lg gels, the gel point temperature (crossover of G′ and G″) was reached at 76.7 ± 0.1 °C, whereas for 10 mM β-lg gels a holding time of 2.9 ± 0.9 min at 90 °C was necessary after the maximum temperature of the heat ramp has been reached (Figure 2). Electrostatic interactions play a significant role in the gelation of whey proteins, such as β-lg. By varying the pH value and ionic strength, electrostatic forces, and therefore, the gelling mechanism and the resulting gel properties can be influenced [19]. The 10 mM β-lg samples formed fibrillar networks and the gelation occurred at higher temperatures [18,40]. At 100 mM a particle network was formed. The difference in the gelation is caused by the electrostatic shielding of the salt ions. Without heat denaturation, β-lg molecules in 10 mM repel each other due to predominant electrostatic interactions, whereas in 100 mM an equilibrium between attractive and repulsive electrostatic interactions is reached [1]. The heat treatment causes the unfolding of the proteins. Therefore, the hydrophobic core is exposed and thus enables protein aggregation [41]. At low ionic strengths, the repulsive electrostatic interactions between the negatively charged β-lg molecules must be overcome, and therefore, the proteins form an extended, fibrillar network and the aggregation occurs at higher temperatures. At high ionic strengths due to charge shielding, the proteins first aggregate into colloidal particles, which further aggregate into fractal gel networks [40,42,43]. Due to the shielding of the negative charges of the protein, the network formation at 100 mM occurred faster and at a lower temperature than the gelation of the 10 mM β-lg gels. 

In the 10 mM mixtures, the onset of gelation (as indicated by the initial increase of G′ during heating) and a constant G′ were reached earlier with increasing levan content. Moreover, the gel point temperature of these mixtures decreased with increasing levan content from 90 °C (+2.9 ± 0.9 min) for 1 wt% Lev4 to 81.4 ± 0.5 °C for 3 wt% Lev4. A further increase of levan content to 5 wt% of Lev4, decreased the gel point temperature only slightly to 80.4 ± 0.1 °C. In the 100 mM mixtures, the gel point temperature shifted to lower temperatures with increasing Lev4 content. However, the differences were less pronounced and the gel point temperatures were generally lower compared to the 10 mM mixtures. Furthermore, the increase of G′ after the onset of gelation was steeper in 100 mM mixtures. The gel point temperature of these mixtures decreased with increasing levan content from 76.7 ± 0.1 °C (0 wt% Lev4) to 75.2 ± 0.3 °C (3 wt% Lev4) and plateaued upon further increase of the levan content. In mixed gels, a decrease of the gel point temperature with increasing content of one polymer is commonly observed if segregative interactions between the polymer species dominate the system [44,45,46]. This type of interaction is caused by thermodynamic incompatibility and is often seen in mixtures of whey proteins and polysaccharides [22,47,48,49,50]. Segregative forces between levan and native β-lg have already been demonstrated and ascribed to the excluded volume effect [1]. Since the excluded volume effect between polymers is affected by the molecular weight, heat-induced aggregation of proteins affects the segregative behavior [7]. As the molecular weight of the protein aggregates increases, the molecular weight difference between the two polymer species increases as well, resulting in more pronounced segregation. The increased segregative forces result in a stronger local accumulation of one of the polymer species, allowing the other species to aggregate and form networks more easily [9]. In our study, this effect was much more pronounced at lower NaCl concentrations. This can be attributed to the shielding of the electrostatic charges. After the heat-induced unfolding of the proteins, aggregation and network formation of the β-lg occurred. At 100 mM, the negative charges of the proteins were largely shielded and attractive molecular interactions allowed a rapid increase of the molecular weight through aggregation and network formation, which could be accelerated only slightly by segregative forces. By contrast, in a 10 mM solution, the electrostatic repulsion of the β-lg molecules had to be overcome after the denaturation of the proteins to enable network formation. In this context, the local accumulation of β-lg in the presence of levan, facilitated the approximation of the repelling proteins, allowing the fibrillar protein network to form more rapidly and at lower temperatures.

The molecular weight of levan affected the heat-induced gelation of the mixed gels depending on the NaCl concentration. In 10 mM solutions, the onset of gelation occurred sooner the higher the molecular weight. The storage modulus of the mixed gels containing the high molecular weight levan Lev4 (2.0 × 10^8^ Da) and Lev5 (6.5 × 10^8^ Da) increased earlier compared to the low molecular weight LevF (1.4 × 10^6^ Da). In addition, a constant value of G′ was reached earlier for the mixtures containing Lev4 and Lev5. The gel point as a function of the molecular weight is displayed in Figure 2 (right panel). For the 10 mM mixtures, a decrease of the gel point from 88.4 ± 0.5 °C (LevF) to 81.4 ± 0.5 °C (Lev4) and 82.0 ± 0.2 °C (Lev5) was observed. By contrast, in the 100 mM mixtures, no effect of the molecular weight on heat-induced gelation was observed. Regardless of the molecular weight, the onset of gelation started slightly earlier if levan was added. The gel point of the 100 mM mixtures dropped by 1.5 to 2.5 °C in the presence of levan (LevF: 74.2 ± 0.0 °C, Lev4: 75.1 ± 0.3 °C, Lev5: 75.2 ± 0.1 °C). The molecular weight dependency of the mixed gels is most likely caused due to the excluded volume effect. At the beginning of the protein aggregation, the levan molecules were significantly larger than the β-lg monomers or dimers. Since the excluded volume effect is more pronounced the higher the molecular weight difference between two polymer species is, the initial effects of segregation were more pronounced the higher the molecular weight of the levan. Therefore, β-lg mixtures with high molecular weight levan formed gels more rapidly and at lower contents compared to mixtures containing low molecular weight levan. This correlation was also shown for other segregative interacting mixed gels [45,46].

### 2.2. Water Binding

Time domain nuclear magnetic resonance (TD-NMR) is a fast and noninvasive tool to study the distribution and mobility of water populations present in food samples [51,52]. The water binding is an important parameter because it can be related to the gel strength of the heat induced β-lg gels. Besides the determined stress (rheology) and network type (SEM), water mobility (NMR) completes the characterization of networks [53].

**Figure 3:**
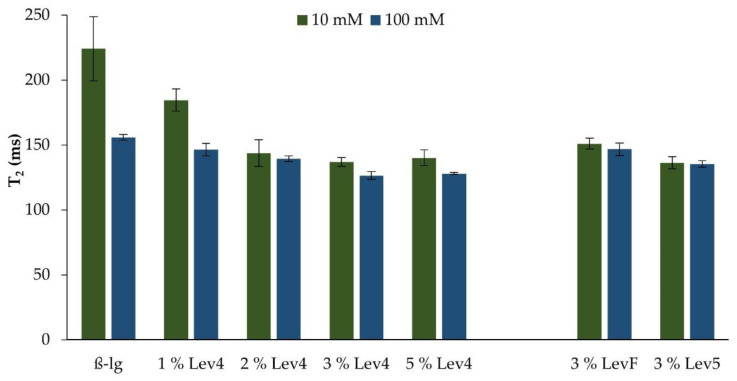
Relaxation times T2 of gels containing different Lev4 contents (**left** side) and different molecular weights of levan (**right** side).

The influence of the salt concentration and the addition of levan on the relaxation times T_2_ of the heat induced β-lg gels can be seen in Figure 3. The relaxation time T_2_ is related to protons (^1^H) with defined rotational mobilities, which can be referred to as the water population within the β-lg gels. An increase in the relaxation time T_2_ means a higher mobility of the water within the gel structure and a less strong water binding. The T_2_ of the 10 mM β-lg gel and the 100 mM β-lg gel indicated a stronger water binding at a higher salt concentration. This correlated with the gel point temperature. The stronger the water binding in the gel was, the lower was the gel point temperature during the heat treatment. Furthermore, propositions on the gel strength can be made by determining the relaxation behavior of the samples, since network formation and water binding are mutually dependent as shown in other studies [54,55,56].

The addition of Lev4 initially decreased the relaxation time T_2_ of the mixed gels at both salt concentrations (Figure 3). The relaxation time of the 10 mM mixed gels decreased with increasing Lev4 content until the addition of 2 wt%. This lower relaxation value was a result of a stronger water binding within the heat induced β-lg gel with increasing levan content. A further increase of the levan content to 3 wt% did not lead to a stronger water binding, although the total polymer content was higher. The mixed 100 mM gels showed similar behavior but less differences in T_2_ with increasing levan content. For the higher salt concentration, the T_2_ value decreased only slightly and remained constant at high Lev4 contents. Almost the same behavior was observed for the gel point temperature in dependency of Lev4 content and salt concentration. This corroborates the hypothesis that the water binding in the mixed gel network corresponds to the network formation, and therefore, with the strength of the segregative behavior of the mixed gels.

On the right-hand side of Figure 3, T_2_ as a measure of water mobility in the gels is shown as a function of the molecular weight of levan. Regardless of the salt concentration, T_2_ of the mixed gels containing 3 wt% high molecular weight levan (Lev4 or Lev5) was higher than T_2_ of the gel containing 3 wt% low molecular weight levan (LevF). At the molecular weight of levan the water molecules are more tightly bound to the gel network resulting in lower mobility of the water protons, which again correlates with the strength of the segregative behavior observed during the heat induced gelation.

### 2.3. Phase Behavior and Network Structure

#### 2.3.1. Rheology

**Figure 4:**
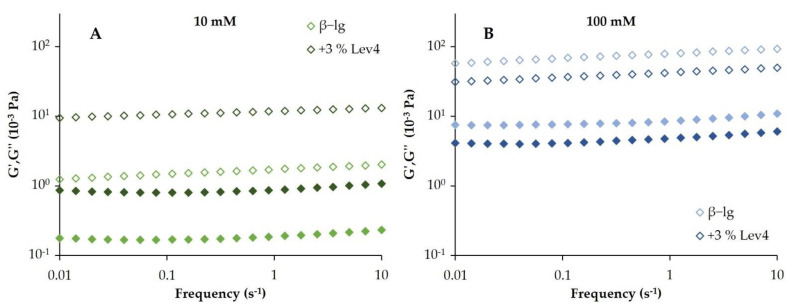
Frequency sweep of the pure β-lg gel (lighter colors) and the β-lg gel containing 3 wt% Lev4 (darker colors) at 10 mM (**A**) and 100 mM (**B**) NaCl. Filled symbols indicate the loss modulus (G″) and open symbols indicate the storage modulus (G′).

After the heating cycle, a frequency sweep was performed to characterize the gels. To ensure that all measurements were performed within the linear viscoelastic range, an amplitude sweep was performed afterward. All rheological tests following the temperature sweep were performed at 20 °C. Exemplarily, G′ and G″ of β-lg and β-lg + 3 wt% Lev4 at 10 and 100 mM are displayed in Figure 4A,B. In addition, G′, G″ and the loss tangent tan(δ) of all gels at a frequency of 0.45 s^−1^ are listed in Table 1. The loss tangent is defined as the quotient of G″ to G′. It can be considered as a measure of the ratio of energy lost to energy stored in the cyclic deformation [57]. In addition, Table 1 contains n, which corresponds to the slope in a double logarithmic plot of G′ versus frequency in the frequency sweep and can be considered as a measure of the frequency dependency of a gel [58]. For a perfectly cross-linked covalent gel, n has a value of 0 while a physical gel has an n > 0. Consequently, the slope is a measure of the similarity of the gel to a covalent gel [18]. All frequency sweeps showed a similar course with G′ > G″ and both modules were largely independent of frequency. Irrespective of the levan addition, the 100 mM particle gels had a significantly higher G′ and G″ than the 10 mM fibrillar gel networks. Both gels showed n values between 0.041 and 0.071 and a tan(δ) between 0.07 and 0.15, indicating strong cross-linked gels. Without levan, the 100 mM gel showed an almost 50-time higher G′ than the 10 mM gel. The G′ of the gels correlated with the water binding observed in the NMR measurements. For the 100 mM β-lg particle gel, a stronger water binding was found compared to the 10 mM fibrillar gel. The correlation between the gel strength (rheology) and the water binding (NMR) was also shown in other studies [54,55,56]. The gel strength of globular proteins is exponentially related to the protein content and depends on pH and ionic strength [59]. For β-lg, this relationship has been demonstrated at pH 7 and increasing NaCl concentration (0–100 mM) [60,61]. This is consistent with the results of our study. We could show that particle gels are associated with a stronger water binding than fibrillar gels. Despite the differences in water binding and network type, both 10 mM and 100 mM gels showed a similar n (0.071) and tan(δ) (0.11). In other studies, a lower n was found for fibrillar gels [18]. This can be explained by influencing factors, such as protein content, heating rate and heating time that may also alter the frequency dependency of the gels.

The addition of Lev4 initially increased the G′ of the mixed gels at both salt concentrations and further decreased after a critical levan content was reached. In 10 mM gels, a maximum of G′ was reached at 3 wt% Lev4. Upon further levan addition, G′ decreased but was still higher compared to G′ of the pure ß-lg gel. Accordingly, tan(δ) and n of the 10 mM mixed gels decreased with increasing Lev4 content and reached their minimum at 3 wt% levan. In 100 mM gels, the maximum of G′ was observed at 2 wt% Lev4. A further increase of the levan content decreased G′ below the value of the pure ß-lg. This indicated a weakening effect of levan in the 100 mM gels. In contrast to the 10 mM mixed gels, the mixed 100 mM gels showed almost no difference in tan(δ). Moreover, n decreased hardly with increasing Lev4 content. The initial increase in G′ with increasing Lev4 content was caused by the accumulation of β-lg in the continuous phase, as there is an exponential relationship between the gel strength and protein content [60,61]. The decrease in n and tan(δ) with increasing levan content also indicated increasing network stability. This behavior was caused by the accumulation of β-lg due to the segregative behavior and resulted in an increased number of crosslinks. By forming a two-phase system, the protein content in the β-lg-rich phase increased, and therefore, the stability of the formed network increased. In the content range from 1 wt% to 3 wt%, also T_2_ decreased indicating a stronger water binding of the mixed gel network. With further increasing Lev4 content, the water binding remained constant or slightly decreased despite the higher total polymer content. In this context, it should be mentioned that gelation and segregative phase separation can occur simultaneously and influence each other as well as the water binding within the network [9,47]. The formation of a three-dimensional gel network with a high viscosity can lead to an arrested state and completely prevent the thermodynamically favorable formation of a two-phase system [62]. In our study, this phenomenon could be observed for the 10 mM, 5 wt% Lev4 mixed gel as discussed in the next chapter.

Regarding the effect of the molecular weight of levan in the 10 mM gels, differences between the low molecular weight (LevF) and the two high molecular weights (Lev4 and Lev5) could be observed. The storage modulus of LevF mixed gel was more than three times lower than Lev4 and Lev5 mixed gels. Accordingly, tan(δ) and n of the gel containing LevF showed higher values indicating a less cross-linked gel structure for the low molecular weight levan. A weaker water binding of the LevF gel was also found in the NMR experiments when compared to the gels containing high molecular weight levan. Since the 10 mM mixed gel containing LevF still showed a stronger water binding, a higher G′ and a lower tan(δ) and n compared to the pure β-lg gel, levan had a synergistic effect on gel formation at 10 mM, which was more pronounced at a higher molecular weight. The effect of molecular weight on gel strength could be explained by increased aggregation due to segregation. Since the excluded volume effect is more pronounced with increasing molecular weight, the segregative forces also increased [62]. However, since only the gel with Lev5 had a comparable G′ to the pure β-lg gel, the molecular weight dependency of the gel strength could also be attributed to steric effects. The lower the molecular weight, the more levan molecules can sterically hinder gel formation and reduce gel strength.

#### 2.3.2. Microstructure

To distinguish between phase separated and swollen gels, water binding and rheological measurements are usually not sufficient. To obtain further information, the appearance of the gels can be evaluated and imaging techniques can be used. Therefore, we used SEM imaging to reveal the network structure of mixed gels containing levan and β-lg.

**Figure 5:**
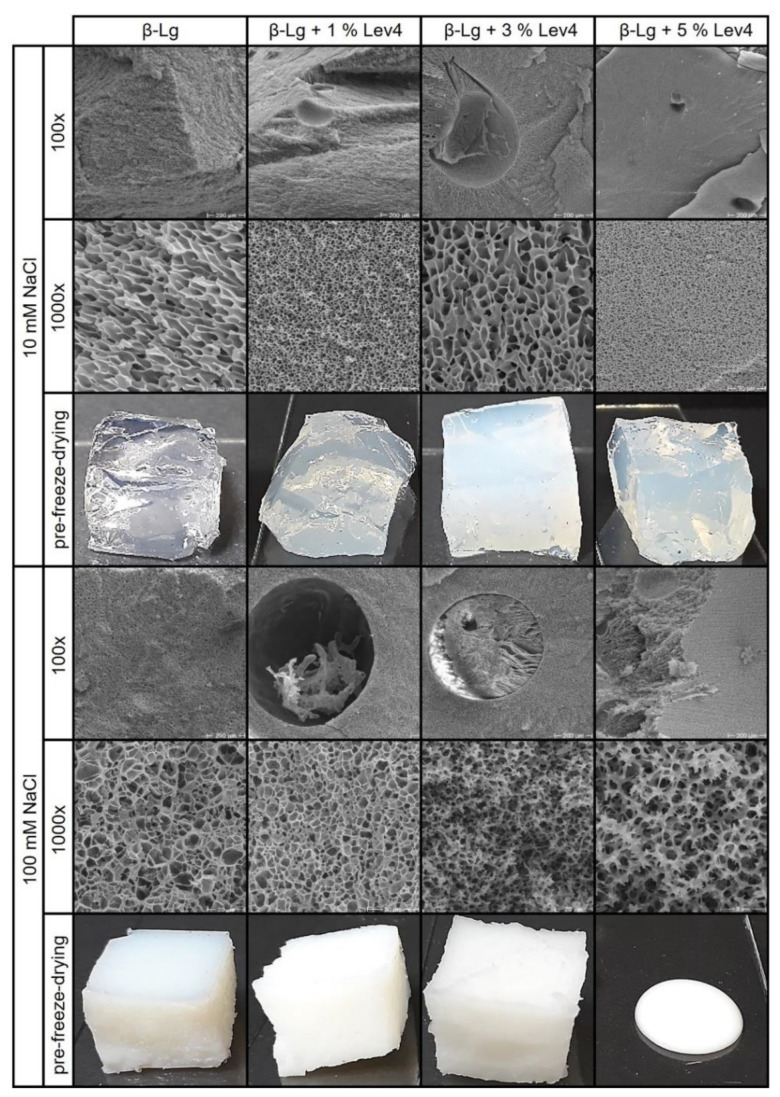
SEM images and photographs of the mixed β-lg gels containing different Lev4 contents in 10 mM and 100 mM NaCl. SEM images at 100× magnification focus on phase-separated regions, if present, and SEM images at 1000× magnification are focused on the con.

The microstructure and the optical images of the β-lg gels and the mixed gels containing Lev4 are shown in Figure 5. SEM images at 1000× magnification display the structure of the continuous β-lg network. The optical appearance can be linked to the gel structure. The fibrillar β-lg gel at 10 mM was transparent since the diameter of the gel strands was in the nm range [20]. A particle network with an opaque appearance was formed at 100 mM because the dimensions of the aggregates were within the µm range [61]. The SEM images of both β-lg gels showed a porous structure that was similar in size but differed in the regularity of the structural elements. It should be noted that the freeze and the freeze-drying process might have affected the structure of the β-lg networks. Therefore, differences between the gels may not only be attributed to the different formulations but also to the alteration of the network structure during preparation.

The SEM images of all Lev4 mixed gels (Figure 5), with exception of 5 wt% Lev4 mixed gel at 10 mM, showed spherical, partial filled inclusions. These spherical inclusions, visible in Figure 5 (100×), differed in structure from the surrounding matrix, indicating segregative phase separation due to thermodynamic incompatibility. This is supported by the fact, that the pore-like structure surrounding the spherical inclusions was comparable to the pure β-lg gels. Therefore, it can be assumed that the filling of the spherical inclusions was a levan-rich phase surrounded by a β-lg-rich continuous phase. Not all spherical structures were filled, which can be explained by the loss of the filling material during the preparation process. A further indication of phase separation in the 10 mM mixed gels, was the increasing turbidity with increasing Lev4 content as the presence of two phases with different refractive indices leads to light scattering. Although one could expect phase separation at a high levan content, the SEM images of the 5 wt% Lev4 mixed gel at 10 mM showed no phase separated inclusions. The 5 wt% Lev4 sample was also less turbid and had a lower G′ than the 3 wt% Lev4 gel. One explanation could be that the viscosity increase caused by levan, may have prevented the separation into two phases [39]. Without the formation of two phases, levan may have led to steric hindrance of gel formation resulting in a decrease in gel strength. Since the 100 mM mixed gels were opaque irrespective of the Lev4 content, the optical evaluation does not provide any information.

Although the network formation of β-lg occurred earlier and faster at high salt concentrations, all SEM images of the 100 mM mixed gels showed inclusions, and therefore, indicate the formation of phase-separated networks. This was even true for 5 wt% Lev4, which did not phase-separate at 10 mM but showed phase separated inclusions at 100 mM. The shape of those inclusions differed from the spherical shape, presumably due to the high viscosity of the sample prior to gelation. The fact that phase separation at 100 mM was not or only partially stopped by gelation—regardless of the levan content—indicated that NaCl enhanced the segregative behavior. In non-heated mixtures of β-lg and levan, it could be shown that phase separation is more likely at a higher NaCl concentration [1]. By shielding the electrostatic charges, the accumulation of β-lg in one phase may have been prevented and made phase separation more likely. The same is true in heat treated systems, in which phase separation is enhanced due to the aggregation of the globular proteins [7]. In contrast to the 10 mM mixed gels (which had a higher G′ than the β-lg gel regardless of the Lev4 content), the G′ of the 100 mM mixed gels decreased below the β-lg gels with 3 wt% or more Lev4. Additionally, n and tan(δ) were merely affected by the Lev4 content at 100 mM. Therefore, segregation supported the gelation of the fibrillar gel (10 mM), while antagonistic effects occurred in the particle gels (100 mM). In 100 mM gels, the accumulation due to the segregation had less impact, since particle gels were already dense due to electrostatic shielding. Especially at higher polymer contents, the system might have been in an arrested state due to the higher viscosity prior to gelation and faster network formation at 100 mM. This resulted in a more homogeneous distribution of levan within the β-lg-rich phase. Consequently, the formation of a coherent network in the β-lg-rich continuous phase was impaired causing G′ to decrease. The homogeneous distribution of Lev4 in the β-lg-rich phase can also explain the structural change of the continuous β-lg phase in the SEM images of the 100 mM mixed gels containing ≥ 3 wt%Lev4.

**Figure 6:**
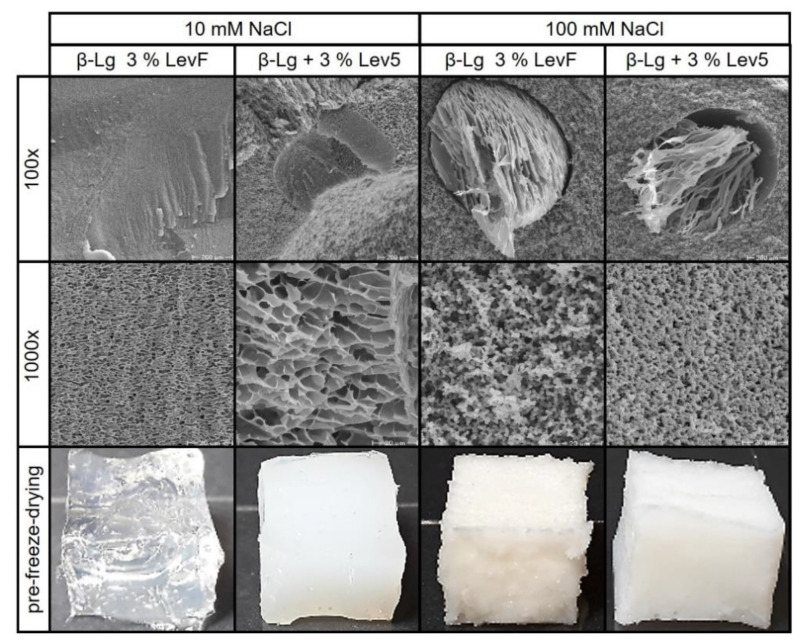
SEM images and photographs of the mixed β-lg gels containing levan of different molecular weight in 10 mM and 100 mM NaCl. SEM images at 100× magnification focus on phase-separated regions, if present, and SEM images at 1000× magnification are focused on the continuous β-lg network.

The molecular weight of levan also influenced the phase behavior during gelation. The addition of the low molecular weight LevF resulted in transparent gels without inclusions at 10 mM, whereas the two high molecular weight levans formed opaque gels with phase separated spherical inclusions (Figure 6). The absence of phase separation in the mixed gel of LevF and β-lg could be due to the smaller size difference between the two polymers. Therefore, the excluded volume effect causes less pronounced segregative forces, which are not sufficient to separate the system into two phases [62]. That segregative forces still acted during gelation, can be seen from the decrease of the gelation temperature and the increase of G′ compared to the pure β-lg gel. However, in contrast to the two high molecular weight levans, the increase in G′ was only marginal. At a salt concentration of 100 mM, G′ increased continuously with the molecular weight of levan. The mixed gel containing Lev5 reached a slightly higher G′ than the pure β-lg gel, while the G′ of the LevF and Lev4 gels were at least two times lower. All three mixed gels showed a tan(δ) of 0.11 and n differed only slightly between 0.068 (LevF, Lev4) and 0.071 (Lev5). The influence of the molecular weight on the gel strength of these gels also corresponded to the strength of the water binding. Despite the higher total polymer content, the T_2_ of the LevF mixed gels was only slightly lower than T_2_ of the 100 mM pure β-lg gel. The mixed gels containing Lev4 and Lev5 exhibited stronger water binding than the pure β-lg gel. The 100 mM mixed gels had an opaque appearance and the SEM images showed phase separated inclusions regardless of the molecular weight. The continuous phase of these gels differed from the pore-like structure, which indicates that levan interfered with the formation of the β-lg particle network at this NaCl concentration regardless of its molecular weight.

## 3. Conclusions

The water binding, rheological and structural properties of heat induced β-lg gels and mixed gels containing various levan contents and molecular weights were studied at two different NaCl concentrations. It was observed that levan enhances the aggregation and gel formation of β-lg. Due to the presence of levan, the water binding of the gel network increased, the onset of gelation occurred earlier and the gel point decreased. This was caused by segregative forces due to the excluded volume effect, which was more pronounced at lower NaCl concentrations, higher levan contents and higher levan molecular weights. Under numerous conditions, a phase-separated gel was formed during the heat treatment. Only at a low NaCl concentration (10 mM), mixed gels with no phase separation could be formed. Here, the repulsion due to unscreened negative charges prevented the local accumulation of protein and the formation of a separated phase. For low molecular weight levan, the segregative forces due to the excluded volume effect were not sufficient to cause phase separation. Furthermore, the high viscosity caused by levan, could have prevented phase separation at the highest polysaccharide content due to an arrested state. While segregative forces based on the presence of levan enhanced network formation, and therefore, increased the gel strength of all 10 mM mixed gels, levan had also antagonistic effects on the 100 mM mixed gels. As the polymer content increased, the gel strength decreased and the mixed gels became weaker than the pure β-lg gel. However, these effects could be compensated by increasing the molecular weight of levan.

This study showed that levan can be used to influence the gelation of β-lg and modify the properties of the resulting mixed gels. Furthermore, NaCl concentration, polysaccharide content and molecular weight can be used to control phase separation, gel strength, optical appearance and water binding. Therefore, our results are a first step toward understanding the behavior of levan in complex food systems. In the future, these results can be transferred to complex food matrixes containing the functional ingredient levan in order to tailor the structure, texture and appearance of food systems.

## 4. Materials and Methods

### 4.1. β-Lactoglobulin and Levan Production

Bovine β-lactoglobulin (β-lg) was isolated from whey protein isolate (Bipro, Agropur Diary Cooperative Inc., MN, USA). The isolation followed the procedure by Keppler et al., 2014 with slight modifications [63]. Ultrafiltration was replaced by dialysis against distilled water for 3 days using BioDesingnDialysis Tubing^TM^ (Thermo Fischer Scientific, Waltham, MA, USA) with a molecular weight cut-off of 14 kDa. Prior to freeze-drying, the pH of the β-lactoglobulin solution was adjusted to pH 7 using 10% HCl (Carl Roth GmbH, analytical-grade, Karlsruhe, Germany).

The acetic acid bacteria *G. albidus* was used to produce three levan samples with different molecular weights. A low molecular weight levan (LevF) was produced by fermentation of a sucrose containing sodium gluconate medium. Two high molecular weight levan samples (Lev4, Lev5) were produced in a cell-free, an enzyme containing sodium acetate buffer at pH 4 and pH 5, respectively. Both levan production methods are described in [38]. The molecular weight of LevF, Lev4 and Lev5 were 1.4 × 10^6^ Da, 2.0 × 10^8^ Da and 6.5 × 10^8^ Da, respectively [38]. The dry mass of β-lactoglobulin and levan was determined with an infrared dryer (LP16, Mettler-Toledo GmbH, Greifensee, Switzerland).

### 4.2. Sample Preparation

All analyzed samples were prepared in 10 mM and 100 mM NaCl solution and contained 10 wt% β-lactoglobulin. To investigate the influence of the levan content on the gelation of β-lactoglobulin, five samples with Lev4 at contents ranging from 0 wt% (*w*/*w*) to 5 wt% (*w*/*w*) were prepared. The influence of the molecular weight was analyzed using levan samples LevF, Lev4 and Lev5 at a polysaccharide content of 3 wt% (*w*/*w*). To prepare the solutions, levan and β-lactoglobulin were weighed in a beaker, mixed with the respective NaCl solution and dissolved on a magnetic stir plate at 300 rpm. Subsequently, the pH value of the solution was adjusted to 7 using 100 mM HCl or 100 mM NaOH solution (Carl Roth GmbH, analytical-grade, Karlsruhe, Germany). To ensure complete hydration, all samples were stored for 14 h in a refrigerator. Prior to the measurements, the pH value was checked and adjusted if necessary.

### 4.3. Rheological Measurements

All rheological measurements were carried out in triplicate using the rheometer Physica MCR 102 or Physica MCR 301 from Anton Paar GmbH (Graz, Austria) equipped with a concentric cylinder system CC27 (Anton Paar GmbH, Graz, Austria). The samples were covered with a layer of paraffin oil and a protective hood was used to prevent evaporation. All experiments were carried out at a frequency of 1 Hz and a strain of 1% unless otherwise specified. Before each measurement, the samples were equilibrated at 20 °C. The gels were formed by submitting the samples to the following thermal cycle: heating from 20 °C to 90 °C at a constant rate of 1 °C/min, holding at 90 °C for 20 min, cooling from 90 °C to 20 °C at a constant rate of 1 °C/min, holding at 20 °C for 10 min. Afterward, a frequency sweep over the range of 0.01 Hz to 10 Hz was carried out at 20 °C. Finally, a strain sweep test was performed from 0.1% to 100% to confirm the experiment was conducted in the linear viscoelastic region. The calculation of n and the gel point temperature was performed using the RheoCompas (Anton Paar GmbH, Graz, Austria) software. The gel point temperature was chosen as the temperature at which G′ and G″ intersect during heating. For samples that gelled after reaching 90 °C, the holding time to attain the intersection of G′ and G″ was also reported.

### 4.4. Scanning Electron Microscopy (SEM)

To produce gels for SEM imaging the solutions were prepared according to the procedure described in Section 2.2. For gelation, the samples were heated in a water bath at 90 °C for 30 min. After cooling, cubes with an edge length of 1 cm were cut from the gels. To observe the optical effects of the thermally induced gel formation, photos were taken after gelation. For freezing the samples were immersed in liquid nitrogen. The sample 100 mM, 5 wt%, Lev4 did not gel during the heat treatment. Therefore, it was placed in a container made of aluminum foil and frozen therein. Immediately after freezing, the samples were dehydrated by freeze drying (Beta 1–8 LSCplus, Martin Christ Gefriertrocknungsanlagen GmbH, Osterode, Germany). Lyophilized samples were carefully broken into pieces and the breakage site was gold sputtered in a sputter coater SCD 030 (Balzers, Wiesbaden-Nordenstadt, Germany). SEM imaging was carried out at the Center for Electron Microscopy (ZELMI, Technische Universität Berlin, Berlin, Germany) with an S-2700 scanning electron microscope (Hitachi, Tokyo, Japan). Images were recorded at a magnification of 100×, 300× and 1000×. SEM was carried out at least on time for each formulation.

### 4.5. NMR

The water binding strength of the heat induced β-lg gels was analyzed using NMR relaxation measurements (time domain (TD)-NMR, Minispec 20 MHz, Bruker Biospin GmbH, Ettlingen, Germany). The spin-spin or “transverse” relaxation time T_2_, and the rate of decay of transverse magnetization, depend on the interaction of the nuclear spins with their environment. Fast molecular movements of the molecules carrying the nuclei result in short interaction times with their neighbors and lead to a slower relaxation process. Transverse relaxation times T_2_ were recorded using the software application “t2_cp_mb”, a Carr–Purcell–Meiboom–Gill (CPMG) pulse sequence provided by Bruker. For each measurement, 4500 data points were collected. Pulse separation between 90° and the 180° pulse was 1 ms and the recycle delay was set to 8 s. Data were accumulated with eight scans. The measurement temperature was 20 °C and measurements were performed at least in triplicate.

To calculate the transversal relaxation time a mono-exponential function was fitted to the NMR intensity (I) data with the fitting toolbox of MATLAB R2019a (The Mathworks Inc., Natick, MA, USA):

(1) $$I=A\cdot e^{-\frac{t}{T_{2}}}$$

where ***I*** is the NMR intensity (%), ***t*** is the measurement time (ms), ***A*** is the amplitude at time zero (%) and ***T*_2_** is the transversal relaxation time of protons (ms).

Inverse Laplace transform was applied to NMR data to obtain the distribution of transverse relaxation times T_2_ (MATLAB R2019a (The Mathworks Inc., Natick, MA, USA)). For inverse Laplace transform, the application (rilt) regularized inverse Laplace transform [g,yfit,cfg] = rilt (t,y,s,g0,alpha) was used [64].

The quality of each fitting was judged based on the sum of the error squares (SSE) and the coefficient of determination (R^2^).

## Figures and Tables

**Table 1:** Storage modulus (G′), Loss modulus (G″), tan(δ) and n of the heat induced β-lg with different levan contents or with different levan molecular weights during the frequency sweeps.

Sample	G′_ω_ = 0.45 (10^−3^ Pa)	G″_ω_ = 0.45 (10^−3^ Pa)	tan(δ)_ω_ = 0.45 (-)	n (-)
β-lg 10 mM	1.6 ± 0.3	0.18 ± 0.01	0.11 ± 0.02	0.071 ± 0.009
+1% Lev4	3.6 ± 0.7	0.30 ± 0.02	0.09 ± 0.02	0.054 ± 0.008
+2% Lev4	5.1 ± 0.1	0.38 ± 0.01	0.07 ± 0.00	0.048 ± 0.000
+3% Lev4	11.4 ± 1.2	0.83 ± 0.11	0.07 ± 0.00	0.047 ± 0.001
+5% Lev4	6.8 ± 0.4	0.51 ± 0.03	0.08 ± 0.00	0.048 ± 0.000
+3% LevF	2.9 ± 0.5	0.25 ± 0.03	0.09 ± 0.00	0.055 ± 0.004
+3% Lev5	9.5 ± 1.0	0.64 ± 0.09	0.07 ± 0.00	0.041 ± 0.001
β-lg 100 mM	75.6 ± 15.4	8.06 ± 1.76	0.11 ± 0.00	0.071 ± 0.001
+1% Lev4	115.5 ± 1.6	13.28 ± 0.15	0.12 ± 0.00	0.071 ± 0.001
+2% Lev4	122.7 ± 1.4	14.13 ± 0.32	0.12 ± 0.00	0.070 ± 0.000
+3% Lev4	40.0 ± 7.5	4.54 ± 0.83	0.11 ± 0.00	0.068 ± 0.000
+5% Lev4	22.0 ± 0.9	2.40 ± 0.09	0.11 ± 0.00	0.068 ± 0.000
+3% LevF	33.5 ± 1.4	3.59 ± 0.16	0.11 ± 0.00	0.068 ± 0.000
+3% Lev5	82.5 ± 11.3	9.18 ± 1.35	0.11 ± 0.00	0.071 ± 0.000

## Data Availability

The data in this study are available on request from the corresponding author.
